# Supplementary material for: A nanoscale view of the origin of boiling and its dynamics
Source: Nat Commun. 2023 Oct 13;14:6428. doi: 10.1038/s41467-023-41959-3 (PMC10576093; doi:10.1038/s41467-023-41959-3)
Supplement: Supplementary file 1 — Supplementary Information. [file 41467_2023_41959_MOESM1_ESM.pdf]

Supplementary Information
A nanoscale view of the origin of boiling and its dynamics

Mirko Gallo[1,2*], Francesco Magaletti[2], Anastasios Georgoulas[2],
Marco Marengo[2,3], Joel De Coninck[2], Carlo Massimo Casciola[1]
1 Sapienza University of Rome, Rome, IT
2 School of Architecture, Technology and Engineering,
University of Brighton, Lewes Road, Brighton, UK
3 Dept. of Civil Engineering and Architecture, University of Pavia, Pavia, IT and
** Corresponding Author: Mirko Gallo. Email: mirko.gallo@uniroma1.it*
(Dated: September 21, 2023)

NUMERICAL SCHEME

The system of Equations 4 can be written in a compact form as

$$\frac{\partial \mathcal{U}}{\partial t} = \mathcal{N}[\mathcal{U}] + \mathcal{K}\mathcal{W} \ , \qquad (1)$$

where $\mathcal{U} = (\rho, \rho\mathbf{u}, \mathbf{T})$ is the state vector collecting the density, momentum, and temperature fields. The non-linear operator $\mathcal{N}$ returns the deterministic dynamics of the system when applied to $\mathcal{U}$. The last term on the RHS of Equation 1 represents the stochastic part of the dynamics. The latter is composed of the non-linear operator $\mathcal{K}$ acting of the stochastic processes $\mathcal{W}$, white noise with zero mean and delta correlation in space and time. The system of Equations 1 has been discretized following the method of lines, which consists of two steps. The first one concerns spatial discretization while, in the second step, the evolution in time is performed. Both of them are crucial for the correct reproduction of the statistical properties of the fluctuating fields at the discrete level. In particular, the adopted numerical scheme must preserve the fluctuation-dissipation balance. This means that the mathematical properties of the relevant continuum differential operators are conserved in the discrete formulation [1], i.e. the gradient operator $\nabla^\dagger = -\nabla\cdot$ should be skew-adjoint, while the Laplacian $\nabla^{2\dagger} = \nabla^2$ self-adjoint. This is the case with central staggered schemes. In fact, by denoting with $\mathcal{G}$ the matrix representing the discrete version of the gradient operator, and with $\mathcal{D}$ the discrete divergence operator, it can be shown that [1]

$$\mathcal{G}^{\mathbf{T}} = -\mathcal{D} \,, \qquad (2)$$

so that the Laplacian is $\mathcal{L} = \mathcal{D}\mathcal{G}$, which is by definition a symmetric (self-adjoint) matrix. In this setting, the discretized fields $\mathbf{U}_n = (\rho_n, \rho\mathbf{u}_n, T_n)$ represents the space-average of the continuum fields over the n-th numerical cell of volume V_n

$$\mathbf{U}_n\,(t) = \frac{1}{\Delta V}\int_{V_n} \mathcal{U}\,(\mathbf{x},\mathbf{t})\ \mathrm{dV} \ . \qquad (3)$$

The discretized version of Equations 1 takes the form

$$\frac{\mathrm{d}\mathbf{U}_{\mathrm{n}}}{\mathrm{dt}} = \mathbf{N}_{nm}\mathbf{U}_m + \mathbf{K}_{nm}\mathbf{W}_m \ , \qquad (4)$$

a set of stochastic ordinary differential equations, where $\mathbf{N}_{nm}\mathbf{U}_m$, and $\mathbf{K}_{nm}\mathbf{W}_m$ are the discrete version of the continuum terms $\mathcal{N}[\mathcal{U}]$ and $\mathcal{K}\mathcal{W}$, respectively. For integration in time, we adopt an explicit second-order Runge-Kutta method, as it is particularly indicated for stochastic integration [2].

SIMULATION SETUP

The set of Equations 4 in the main text is numerically solved with appropriate boundary conditions. All the simulations are performed in a cubic domain with a side length L discretized with cubic cells with volume Δ^3 ($L^3 = N_c\Delta^3$), with N_c the total number of cells. For all the simulations, the adopted grid spacing is $\Delta = 6$, and the numerical time step is $\Delta t = 0.1$. The direction $\mathbf{z}$ is perpendicular to the wall, i.e. $z = 0$ is the wall location and $z = L$ is the top of the numerical domain. In the other two directions parallel to the solid wall surface, $\mathbf{x}$, $\mathbf{y}$, periodic boundary conditions are imposed. Boundary conditions on the solid wall and upper domain boundary are discussed below. In Supplementary Figure 1 a two-dimensional section of the numerical setup is reported. The fluid initially at saturation conditions is progressively heated with an imposed heat flux Q at the solid wall, *i.e.* $-\mathbf{q}\cdot\hat{\mathbf{n}} = Q$ ($Q > 0$ means heating the fluid), with $\hat{\mathbf{n}}$ the outwards normal and $\mathbf{q}$ the energy flux vector.

In pool boiling, far from the heated wall, the liquid is kept at saturation, hence pressure and temperature are prescribed on the top of the numerical domain. On the wall, an externally imposed heat flux heats up the fluid $\mathbf{q}\cdot\mathbf{z} = Q$. Finally, no-slip and impermeable conditions are assigned to the velocity at the wall. The normal derivative of the density field at the wall is related to the solid/fluid energy density Eq.2 in the main text according to

$$\frac{\partial\rho}{\partial z} = \frac{\partial f_w}{\partial\rho} = -\cos\phi\sqrt{\frac{2}{\lambda}\left[\omega_b(\rho,T) - \omega_b(\rho_V)\right]}\,.$$

Non-reflecting boundary conditions are enforced on $z = L$ to avoid unphysical pressure wave reflections at the artificial boundary [3, 4]. The numerical algorithm used is

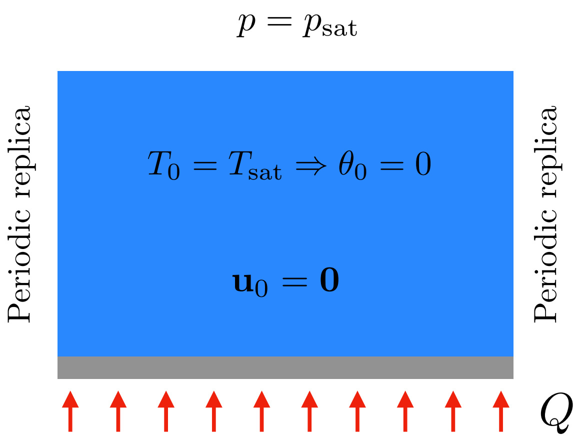

Supplementary Figure 1. Sketch of a two-dimensional section of the numerical experiments.

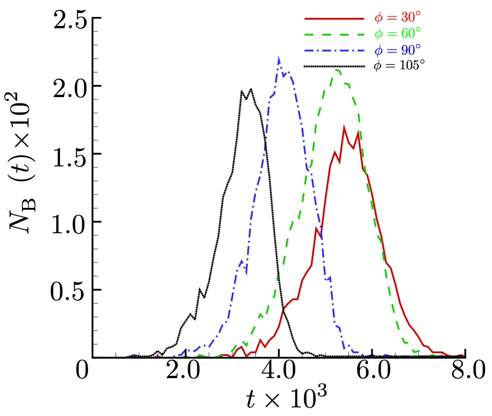

Supplementary Figure 2. Number of supercritical bubbles vs time. Different curves represent the different wettability values.

δ	$Q = 0.001$	$Q = 0.005$	$Q = 0.01$
$\phi = 30°$	1.17	0.63	0.33
$\phi = 60°$	1.12	0.70	0.37
$\phi = 90°$	1.17	0.85	0.73
$\phi = 105°$	1.41	1.08	0.67

Supplementary Table 1. Values of the Tolman length for the different values of the contact angle ϕ and the heat flux Q

described in [5, 6] and consists of a staggered discretization with second-order spatial accuracy and explicit integration in time (second-order Runge-Kutta).

All quantities reported in the text and in the figures are dimensionless, with the reference value chosen as the critical ones (reduced units in Van der Waals EoS). For instance, by considering water, one has $T_c = 647$K for temperature, $p_c = 22$MPa for pressure, $\rho_c = 196.8{\rm Kg}/m^3$ for density. Consistently, $L_R = (k_B T_c/p_c)^{1/3} = 0.74$nm, $u_R = (p_c/\rho_c)^{1/2} = 334.8$m/s, $t_R = L_R/u_r = 2.21$ps, and $q_R = p_c u_r = 7.56{\rm GW/m}^2$. The value of the capillary coefficient is fixed to $\lambda = 5.3 \times 10^{-16}{\rm m}^7/{\rm s}^2{\rm kg}$ to reproduce the correct value of the surface tension of water ($\sigma = 0.072$N/m), providing a liquid/vapor interface thickness at ambient conditions of $\epsilon = 1.3$nm in accordance with experimental observation [7]. Clearly at higher temperatures the surface tension decreases and the interface thickness increases, for example, $\epsilon = 5.8$nm and $\sigma = 0.0025$N/m when $T/T_c = 0.95$. Finally, the transport coefficients, namely viscosities $\eta_1, \eta_2(\rho, T)$ and thermal conductivity $k(\rho, T)$, are taken from the International Association for the Properties of Water and Steam (IAPWS). [8].

NUMBER OF BUBBLES ON ULTRA-SMOOTH WALLS

In order to identify the number of supercritical bubbles in the field we use clustering algorithms. The first step consists of a cell-flagging procedure where all volume cells containing the vapor phase are selected. The vapor non-dimensional density threshold is selected as $\rho_c = 1$, which is the critical Van der Waals density. The cluster analysis is then applied to all the colored cells. By analyzing neighbouring cells of a given vapor cell, the algorithm is able to identify distinct vapor embryos. Among the entire list of clusters, we only select the ones that are supercritical. The critical size of the bubbles is evaluated in [6], where the effective critical radius, is associated with a transition probability of 50%. So, the probability of becoming a macroscopic bubble for a given radius is 1/2 the iso-committor probability in transition state theory. The clustering algorithm is invoked at each time instant. In Supplementary Figure 2, the number of supercritical bubbles $N_{\rm B}^{\rm SC}(t)$ is reported as a function of time. Different curves refer to different values of the contact angle, as indicated in the legend.

TOLMAN LENGTH VALUES

The Tolman lengths used for the CNT correction are provided in Supplementary Table 1 (see the methods section in the main text).

[1] F. Balboa, J. B. Bell, R. Delgado-Buscalioni, A. Donev, T. G. Fai, B. E. Griffith, and C. S. Peskin, Multiscale Modeling & Simulation **10**, 1369 (2012).

[2] S. Delong, B. E. Griffith, E. Vanden-Eijnden, and A. Donev, Physical Review E **87**, 033302 (2013).

[3] T. J. Poinsot and S. Lele, Journal of Computational Physics **101**, 104 (1992).

[4] R. Delgado-Buscalioni and A. Dejoan, Physical Review E **78**, 046708 (2008).

[5] M. Gallo, F. Magaletti, and C. M. Casciola, Physical Review Fluids **3**, 053604 (2018).

[6] F. Magaletti, A. Georgoulas, and M. Marengo, International Journal of Multiphase Flow **130**, 103356 (2020).

[7] F. Caupin, Physical Review E **71**, 051605 (2005).

[8] J. Kestin, J. Sengers, B. Kamgar-Parsi, and J. L. Sengers, Journal of Physical and Chemical Reference Data **13**, 175 (1984).
